# Single-Cell and Multi-Omics Profiling of the Multiple Myeloma Immune Niche: From Bone Marrow Architecture and Metabolic Reprogramming to Immunotherapy Response

**DOI:** 10.3390/cancers18193158

**Published:** 2026-09-29

**Authors:** Katia Beider, Olga Ostrovsky, Arnon Nagler

**Affiliations:** 1 Department of Hematology and Bone Marrow Transplantation, Chaim Sheba Medical Center, Tel-Hashomer 52621, Israel; 2 Jerusalem Multidisciplinary College, Jerusalem 9101001, Israel

**Keywords:** multiple myeloma, single-cell RNA sequencing, multi-omics, bone marrow microenvironment, immune niche, spatial transcriptomics, immunotherapy, metabolic reprogramming

## Abstract

Multiple myeloma (MM) is increasingly understood as a spatially organized and dynamically evolving disease, in which malignant plasma-cell behavior is shaped by local immune, stromal, vascular, and metabolic contexts. Single-cell, spatial, and multi-omic technologies have resolved tumor-cell heterogeneity, bone marrow niche remodeling, immune dysfunction, and therapy-induced selection at unprecedented resolution, yet an integrated synthesis linking marrow architecture, extramedullary dissemination, circulating tumor cells, metabolic reprogramming, chimeric antigen receptor T (CAR-T) cell and T-cell-redirecting immunotherapy, and stem-cell transplantation remains lacking. Here, we review how malignant subclones occupy distinct marrow regions, focal lesions, and extramedullary sites with different transcriptional, antigenic, immune-interactive, and metabolic states. We discuss how spatial transcriptomics and multiplexed imaging reveal immune exclusion, stromal support, oxidative and glycolytic niches, and region-specific antigen heterogeneity. We further examine how circulating tumor cells serve as liquid readouts of tumor burden, high-risk genomics, and clonal evolution. Emphasis is placed on CAR T-cell and T-cell engager therapies, where response and resistance depend on antigen retention, effector-cell fitness, clonal T-cell expansion, myeloid suppression, and spatial accessibility. Finally, we consider autologous hematopoietic stem-cell transplantation as a clinical model of cytoreduction, lymphodepletion, and incomplete immune reconstitution. We propose that MM progression and relapse should be interpreted through an integrated spatial-temporal framework, in which therapeutic outcome reflects the interaction between malignant plasma-cell plasticity, immune competence, stromal persistence, metabolic adaptation, and residual disease localization.

## Simple Summary

Multiple myeloma is a spatially organized disease shaped by immune, stromal, and metabolic niches. Immune dysfunction progresses across MGUS, SMM, MM, and refractory disease and is coordinated by cell–cell crosstalk, cytokines, and extracellular vesicles. Single-cell and spatial multi-omic platforms (Visium, CosMx, GeoMx DSP, CODEX, IMC) are complementary tools for interpreting the multiple myeloma niche. CTCs mirror bone-marrow biology and predict adverse outcome, but do not replace tissue-based spatial analysis. Metabolic reprogramming shapes plasma-cell survival and constrains T-cell and CAR T-cell fitness in the marrow niche. Autologous HSCT is a cytoreductive and immunomodulatory intervention producing incomplete immune reconstitution and a conditional therapeutic window for consolidation. We propose an “immune-fitness” framework, interpreted in spatial context, to stratify patients for CAR T-cell, bispecific-antibody, and combination immunotherapy.

## 1. Introduction

Multiple myeloma (MM) is a malignancy of terminally differentiated B cells characterized by the accumulation of transformed plasma cells (PCs) in the bone marrow (BM). In almost all cases, MM develops from the asymptomatic precursor conditions monoclonal gammopathy of undetermined significance (MGUS) and smoldering myeloma (SMM) [1]. Therapeutic advancements, including proteasome inhibitors, immunomodulatory agents, anti-CD38 monoclonal antibodies, chimeric antigen receptor -T (CAR -T) cell and T-cell redirecting immunotherapies, have significantly improved patient outcomes and may even cure the disease in a selected group of patients. Nevertheless, MM remains difficult to treat due to profound inter-patient and intra-tumor heterogeneity [1,2,3]. Malignant PCs differ in their genomic alterations, subclonal composition, transcriptional programs, surface antigen expression, and treatment sensitivity. Moreover, a growing body of evidence indicates that a reciprocal relationship between the tumor and the BM niche sustains tumor growth and affects treatment responses. Immune cells, mesenchymal stromal cells, vascular components, and osteolineage populations contribute to PC survival and can promote immune suppression and therapeutic resistance [4].

**Figure 1:**
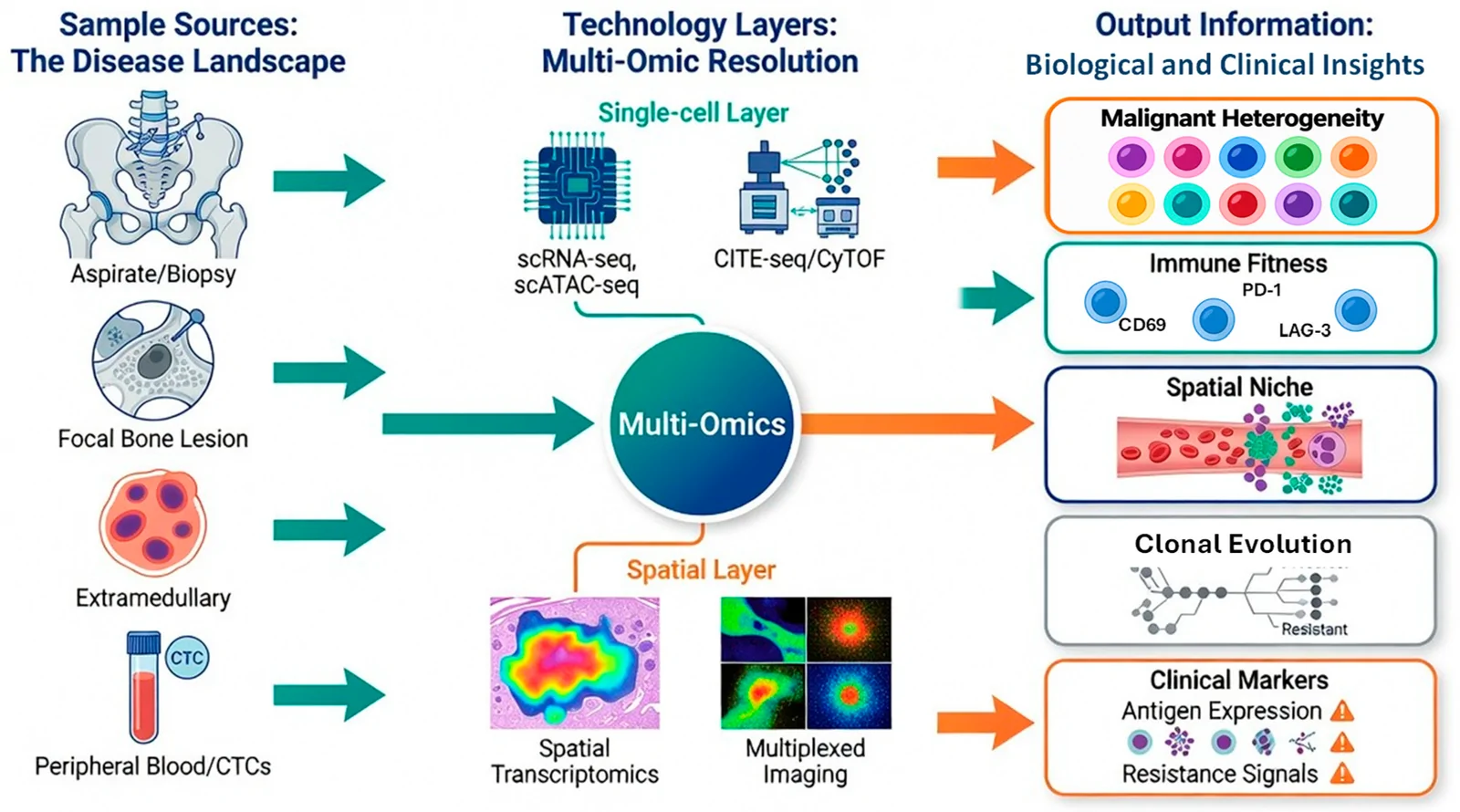
Integrated single-cell, spatial, and multi-omic profiling of multiple myeloma across anatomical compartments. Schematic overview of how complementary sampling strategies and high-resolution molecular technologies can be integrated to characterize the biological and clinical heterogeneity of multiple myeloma (MM). Sample sources encompass bone marrow aspirates and biopsies, imaging-guided focal bone lesions, extramedullary disease sites, and peripheral blood containing circulating tumor cells (CTCs), thereby capturing disease across distinct anatomical and spatial compartments. These specimens can be interrogated through complementary single-cell and multi-omic approaches, including single-cell RNA sequencing (scRNA-seq) to define transcriptional cell states, single-cell assay for transposase-accessible chromatin sequencing (scATAC-seq) to characterize chromatin accessibility and regulatory programs, and protein-integrated single-cell technologies such as cellular indexing of transcriptomes and epitopes by sequencing (CITE-seq) and cytometry by time-of-flight (CyTOF). Integration of these molecular layers provides a multidimensional representation of malignant and non-malignant cell populations. In parallel, spatial transcriptomics and multiplexed imaging preserve tissue architecture and resolve the localization, proximity, and cellular interactions of malignant plasma cells, immune populations, and stromal components within the bone marrow and other disease sites. Together, these approaches generate several interconnected levels of biological and clinically relevant information, including identification of malignant cellular and molecular heterogeneity, assessment of immune fitness and functional immune states, including activation and exhaustion-associated features such as CD69, PD-1, and LAG-3 expression, characterization of spatially organized tumor–microenvironment niches, reconstruction of clonal architecture and evolutionary trajectories associated with disease progression and therapeutic resistance, and identification of clinically relevant biomarkers such as variable therapeutic-antigen expression and resistance-associated molecular programs. Integration of these complementary data layers provides a framework for understanding MM as a spatially and molecularly heterogeneous disease and for identifying features associated with progression, immune dysfunction, therapeutic vulnerability, and treatment resistance. Abbreviations: MM, multiple myeloma; CTCs, circulating tumor cells; scRNA-seq, single-cell RNA sequencing; scATAC-seq, single-cell assay for transposase-accessible chromatin sequencing; CITE-seq, cellular indexing of transcriptomes and epitopes by sequencing; CyTOF, cytometry by time-of-flight; PD-1, programmed cell death protein 1; LAG-3, lymphocyte activation gene 3.

Traditional genomic analyses often fail to discover the full complexity of MM. Bulk and multi-region sequencing first revealed extensive intra-clonal genetic diversity and spatially different sub-clonal populations across separated sites of BM involvement, including focal lesions [2,3,5]. The advent of single-cell RNA sequencing (scRNA-seq) and spatial transcriptomics extended this picture, resolving PC heterogeneity across the MGUS-SMM-MM continuum, demonstrating that programs governing proliferation and cellular metabolism intensify progressively with disease stage [6,7]. Single-cell studies further showed that subclones interact differentially with their microenvironment, shaping an immunosuppressive BM milieu during relapse and refractoriness [8]. scRNA-seq has shown that immune surveillance is compromised already at precursor stages and that the tumor undergoes clonotypic and transcriptional evolution before symptomatic disease [9,10]. More recently, integration of single-cell transcriptomic, multi-omic, and spatial approaches has enabled simultaneous characterization of malignant cells and their surrounding cellular components and has identified candidate interactions with potential therapeutic relevance [11,12]. Furthermore, spatial transcriptomics has begun to determine where tumor and immune populations are positioned within the intact BM tissue [13]. In parallel, integrated single-cell analyses have linked specific immune cell states and microenvironment features with clinical outcomes and with response or resistance to T cell redirecting therapies [14,15] (Figure 1).

In this review, we discuss recent advances in single-cell, spatial, and multi-omic analyses of MM. We examine how these approaches have refined our understanding of malignant PC heterogeneity, spatial organization of the BM niche, changes in stromal and immune compartments, and metabolic adaptation during disease progression. We also consider how these features influence response and resistance to CAR T-cell therapy and T cell-engager immunotherapies. Together, these studies indicate that MM progression and relapse reflect coordinated changes in malignant cells and their surrounding microenvironment rather than tumor cell evolution alone. Particular attention is given to metabolic changes within the BM niche and their potential contribution to immune dysfunction and therapeutic resistance.

## 2. Immune Dysregulation in the MM Microenvironment

The immune BM microenvironment composition in MM has been identified as a factor affecting tumor progression and therapeutic outcomes. Immune dysfunction in MM is not an end-stage phenomenon but a progressive process: alterations of T cell, NK cell, and myeloid compartments are already detectable in MGUS, become more pronounced in SMM and symptomatic MM, and intensify further at relapse and in refractory disease [9,10,16].

T cell dysfunction is one of the central features of the MM immune niche. During progression, cytotoxic T cells may acquire exhaustion-associated programs, with increased expression of inhibitory receptors PD-1, TIM-3, LAG-3, and TIGIT, and impaired cytokine production [9]. Furthermore, T cell senescence, characterized by loss of CD28 co-stimulatory competence, acquisition of CD57, telomere shortening, and reduced proliferative capacity, was detected in BM of MM patients [17,18]. These programs are accompanied by expansion of regulatory T cells (Tregs), which further suppress effector T cell function and increase in frequency with disease progression [16].

Natural killer (NK) cells are essential mediators of both innate anti-myeloma cytotoxicity and antibody-dependent cellular cytotoxicity (ADCC) engaged by monoclonal antibodies such as daratumumab, isatuximab, and elotuzumab. In MM, NK cells frequently show altered maturation, defective adhesion, reduced cytotoxicity, and exhaustion-like features [19,20,21,22].

The myeloid compartment adds a further layer of immune suppression. Myeloid-derived suppressor cells (MDSCs), both granulocytic and monocytic, expand in the BM and peripheral blood of patients with symptomatic MM and suppress T cell and NK cell function through arginase 1, inducible nitric oxide synthase, reactive oxygen species, and PD-L1 [23,24]. Tumor-associated macrophages (TAMs) are consistently skewed toward an M2-polarized immunosuppressive phenotype in the MM niche and provide direct pro-tumoral support, secreting survival factors such as IL-6, BAFF and APRIL, while also contributing to immune suppression through inhibitory cytokines IL-10 and TGF-β, checkpoint-ligand expression, and altered antigen-presentation programs [25,26].

Supporting cells of the marrow further amplify immune escape. Mesenchymal stromal cells can acquire inflammatory and immune-regulatory states that persist despite tumor reduction [27]. Endothelial and vascular compartments influence oxygenation, immune-cell trafficking, and effector-cell access to PC clusters. Osteoclasts are not only mediators of bone destruction, but also immunomodulatory cells. They can suppress proliferating CD4^+^ and CD8^+^ T cells, express inhibitory molecules, produce APRIL, and induce PD-L1 expression on myeloma cells [25,28].

Malignant PCs actively participate in shaping this immunosuppressive niche. They engage surrounding immune and stromal cells through direct ligand-receptor interactions, cytokines, chemokines, and extracellular vesicles. Several signaling axes are particularly relevant. CXCL12-CXCR4 interactions regulate marrow retention and spatial localization. IL-6, BAFF, APRIL, TGF-β, IL-10, and inflammatory cytokine networks support PC survival while restraining effective immunity [25,26,29]. Myeloma- and stroma-derived extracellular vesicles (EVs) add an additional layer of communication. EVs carry immunomodulatory proteins, microRNAs, checkpoint-associated molecules, and metabolites, promoting T cell dysfunction and reshaping the local immune milieu [30,31,32]. Extracellular-vesicle signaling may also affect stromal cells, osteoclasts, and endothelial cells, thereby linking immune suppression to bone destruction, angiogenesis, and niche remodeling [33].

Thus, immune dysregulation in MM is not confined to lymphocytes. It is generated by coordinated crosstalk among malignant PCs, stromal cells, myeloid cells, vessels, and bone-remodeling compartments. The spatial organization of these interactions is critical.

## 3. Single-Cell and Spatial Multi-Omic Platforms Used to Study the Myeloma Niche

Single-cell and spatial technologies provide complementary views of MM biology. Dissociated single-cell methods define the cellular and molecular composition of the tumor and immune microenvironment. Spatial methods preserve tissue architecture and show where these cells are located. Multi-omic approaches integrate several molecular layers, allowing malignant PC states, immune dysfunction, stromal remodeling, antigen heterogeneity, and therapy resistance to be interpreted together.

scRNA-seq is the most widely used platform in MM studies. Droplet-based scRNA-seq enables transcriptomic profiling of thousands of individual cells and has become central for resolving cellular heterogeneity in complex tissues [34]. Its main limitation is the loss of spatial information during tissue dissociation.

Single-cell immune-repertoire sequencing, including B-cell receptor sequencing (scBCR-seq) and T-cell receptor sequencing (scTCR-seq), adds clonal information to transcriptomic data.

Cellular Indexing of Transcriptomes and Epitopes by Sequencing (CITE-seq) and related antibody-oligonucleotide approaches combine single-cell transcriptomics with surface-protein measurement. CITE-seq uses oligonucleotide-labeled antibodies to integrate cellular protein and transcriptome measurements in the same single-cell readout [35]. This is important because RNA expression does not always predict protein abundance, especially for therapeutic targets and immune-checkpoint molecules.

Single-cell assays for transposase-accessible chromatin through high-throughput sequencing (scATAC-seq) measures chromatin accessibility and therefore provides information about gene-regulatory states [36]. In MM, scATAC-seq can help distinguish transcriptionally similar but epigenetically different malignant subclones, identify regulatory programs associated with focal lesions or antigen escape, and define T-cell differentiation states. When combined with scRNA-seq, single-cell multi-omic approaches link chromatin accessibility and gene expression within the same cell, providing a stronger framework for understanding cellular plasticity and therapy-induced adaptations.

Spatial transcriptomics extends single-cell analysis by preserving the tissue architecture in which malignant PCs interact with their microenvironment. Sequencing-based platforms distribute barcoded probes across a tissue section and recover a near-genome-wide readout. The 10x Genomics Visium platform captures polyadenylated or probe-hybridized transcripts on ~55 μm spots [37], while the newer Visium HD assay reduces the effective feature size to a 2 μm grid, typically aggregated to 8 or 16 μm bins for cell-type assignment. NanoString/Bruker GeoMx Digital Spatial Profiler (DSP) takes a distinct, region-of-interest (ROI)-driven approach: fluorescent morphology markers define user-selected areas of illumination, from which UV-cleavable oligonucleotide tags are released and quantified, allowing paired RNA and protein profiling of morphologically defined compartments [38]. Imaging-based platforms resolve individual transcripts and assign them to single cells through membrane- and nucleus-based segmentation. The 10x Genomics Xenium system uses padlock probes and rolling-circle amplification with targeted panels of up to ~5000 genes [39], and the NanoString/Bruker CosMx Spatial Molecular Imager (SMI) uses cyclic in situ hybridization of fluorescent molecular barcodes to profile up to 6000 targets and, more recently, a whole-transcriptome panel, at subcellular resolution [40].

Together, these platforms answer different but complementary questions. scRNA-seq defines cell states, immune-repertoire sequencing identifies clonal relationships, CITE-seq and cytometry validate protein-defined phenotypes, while scATAC-seq and multi-ome approaches reveal regulatory programs. Spatial transcriptomics maps tissue organization, and multiplexed imaging confirms protein-level neighborhoods. In MM, the strongest biological interpretation comes from integrating these layers rather than relying on a single platform.

## 4. The Bone Marrow Niche: Cellular and Spatial Architecture

MM develops within a structurally complex BM environment composed of vascular, stromal, osteolineage, adipocytic, myeloid, lymphoid, and PC compartments that occupy distinct spatial niches. This organization influences cell survival, immune cell distribution, cytokine signaling, nutrient access, and response to therapy [41]. Accordingly, understanding MM requires characterization not only of the malignant PCs present in a sample, but also of their spatial location and interaction with neighboring cells that may promote or limit their growth.

An important basis for this concept comes from recent mapping of normal BM. Bandyopadhyay et al. paired single-cell transcriptomics with CODEX (co-detection by indexing) multiplexed imaging to chart how the human BM is organized in situ, resolving the non-hematopoietic stroma into nine transcriptional subtypes and placing very large numbers of cells in their native tissue context. Marrow populations were found not to be randomly intermixed but to occupy defined territories, among them an oxygen-rich arterio-endosteal region tied to early myelopoiesis and an adipocyte-rich region enriched for early hematopoietic progenitors [42]. Thus, understanding the normal spatial architecture of marrow provides an essential framework for interpreting how disease-associated remodeling supports MM initiation, progression, and microenvironment dependence.

Early genomic studies showed that MM is spatially heterogeneous. Using multi-region sequencing, Rasche et al. demonstrated that anatomically distinct marrow sites and focal lesions from the same patient can harbor different genomic subclones [3]. These observations raised concerns about the ability of a single iliac crest aspirate to represent the full genetic complexity of the disease and suggested that focal lesions may provide local conditions that favor expansion of subclones. Longitudinal studies further supported this interpretation. In an imaging-guided analysis of 24 patients followed for up to 14 years, with whole-exome sequencing of 140 spatially and temporally resolved samples, focal lesions were identified as important sites of clonal evolution. Baseline lesions were phylogenetically close to the clones detected at relapse, and the presence of three or more PET-avid lesions at diagnosis was associated with relapse involving several distinct subclones [43]. These findings suggest that spatial heterogeneity contributes to clonal selection and may promote the development of treatment resistance.

John et al. examined spatial heterogeneity at single-cell resolution by comparing image-guided biopsies with random iliac-crest aspirates from newly diagnosed patients using bulk sequencing, scRNA-seq, and scATAC-seq. The tumors were highly polyclonal, with an average of approximately six subclones per patient across the 17-patient cohort, and several subclones were restricted to focal lesions. Importantly, it was found that spatial tumor heterogeneity was frequent: in most evaluable patients, the dominant subclone within a focal lesion was absent, or present only as a minor population. The study also showed that genetically similar subclones could display different transcriptional states depending on their anatomical location, so that immunotherapy targets such as CD38 and CD20 could be expressed at different levels by the same clone depending on its location [12]. These findings indicate that focal lesions may harbor distinct tumor populations that are not adequately represented by standard iliac crest sampling.

The same study showed that focal lesions differ from random marrow not only in tumor cells but also in the surrounding microenvironment. John et al. reported a significant depletion of macrophages within the focal lesion microenvironment. Macrophages sustain MM cells through several routes, including IL-6/STAT3 signaling, promotion of angiogenesis, and BAFF/BCMA interactions [26]. Their relative scarcity may indicate that some local lesion subclones may become less dependent on marrow support signals. In parallel, the study observed an increased proportion of mesenchymal stromal cells in focal lesion milieu, together with changes in CXCL12/CXCR7 signaling, and only proportional changes in the T cell repertoire without site-specific expansion of T cell clones in intramedullary lesions [12]. The stromal findings dovetail with work by de Jong et al., whose single-cell profiling cast the MM microenvironment as an inflammatory stromal landscape and pinpointed CXCL12-expressing inflammatory mesenchymal stromal cells that nurture MM cells, sit alongside immune populations, and remain present after induction therapy [27].

The functional consequences of the subclonal microenvironment interplay were directly addressed by Tirier et al., who applied single-cell transcriptomics to paired samples from refractory disease and showed that distinct genetic subclones exert subclone-specific effects on their local microenvironment and exhibit differential drug responses, within an immunosuppressive milieu marked by exhausted T cell and altered myeloid populations [8].

The stromal compartment has since been characterized in greater detail. Ju and colleagues performed scRNA-seq of the CD138-negative BM fraction from newly diagnosed MM patients and healthy donors and integrated these data with bulk transcriptomic cohorts. Six mesenchymal populations were identified, including stemness- and proliferation-associated clusters, with osteogenic, angiogenic, immune-regulatory, and multipotent clusters. Their relative abundance changed with disease stage. Osteogenic mesenchymal cells were most prominent in healthy BM and progressively declined with advancing R-ISS stage. Angiogenic mesenchymal cells, by contrast, were enriched in end-stage disease. Immune regulatory mesenchymal cells markedly expanded already in early R-ISS I stage and were associated with poorer overall survival [44]. Within this compartment, the authors identified three transcriptional states: an extracellular matrix remodeling population capable of forming a physical barrier to immune cell and drug infiltration, a stress-adaptive autophagy-associated subset, and a previously undescribed population termed HMGhMSC, defined by expression of high mobility group proteins. The HMGhMSC population expressed immunomodulatory factors and was predicted to interact with several immunne populations. Among the identified signaling pathways were CD44-SPP1 (osteopontin), associated with reduced CD8^+^ T-cell activation and tolerogenic macrophage polarization, and GAS6-AXL, which was linked to regulation of NK cells, dendritic cells, M2 macrophages, and regulatory T cells. From the HMGhMSC transcriptome, the authors built a 20-gene signature that stratified patient survival independently of ISS [44]. These results show that mesenchymal populations in MM are functionally heterogeneous and that specific stromal states are associated with immune regulation, bone disease, and clinical outcome.

More recent spatial transcriptomic approaches have allowed these cellular relationships to be examined directly in intact BM tissue. Yip et al. applied subcellular-resolution imaging-based spatial transcriptomics (10x Genomics Xenium) using a panel of approximately 5000 genes to trephine biopsies spanning MGUS, SMM, and active MM [13]. This approach enabled malignant and normal PCs, stromal cells, and immune populations to be analyzed while preserving their position within the tissue. The study revealed substantial variation in both cellular composition and spatial organization among samples and demonstrated the feasibility of examining the spatial relationships between PCs and stromal or immune populations directly in BM biopsies across different disease stages.

Muinos-Lopez et al. provided a complementary approach by applying Visium spatial transcriptomics to formalin-fixed paraffin-embedded BM sections from healthy and MM mouse models, as well as from patient biopsies. The authors identified spatially restricted pathogenic gene programs, including signatures of NETosis and IL-17 signaling, that were reduced in regions enriched for malignant PCs [45]. This finding suggests that inflammatory activity is unevenly distributed across the marrow and may be locally suppressed in PC-rich areas. The study also identified a shift from effector-like toward exhausted T cell states with increasing distance from PC-rich regions, indicating that immune cell functions in MM are shaped by tissue location as well as by overall disease burden.

Single-cell and spatial studies therefore provide complementary information about immune dysfunction in MM. scRNA-seq defines immune populations according to their transcriptional and functional states, whereas spatial approaches determine where these cells are located in relation to PCs and other components of the BM niche. This distinction is clinically meaningful because the presence of cytotoxic T cells does not necessarily indicate effective antitumor immunity if these cells are excluded from PC-rich regions or are exposed to suppressive stromal or myeloid signals. Evaluation of the MM microenvironment should therefore consider cellular composition, functional state, and spatial organization together with interpreting immune competence and disease behavior.

## 5. Extramedullary Disease and the Spatial Architecture of Myeloma Beyond the Marrow

Extramedullary disease (EMD) is one of the most aggressive manifestations of MM. It occurs when clonal PCs establish tumors outside the BM, either as hematogenously disseminated soft-tissue lesions or through direct extension of skeletal lesions into adjacent tissues. True EMD, in contrast to paraskeletal involvement, reflects the ability of malignant PCs to survive independently of the conventional BM niche. It is associated with high-risk disease, treatment resistance, and poor prognosis, including in patients treated with T-cell-redirecting immunotherapies [46].

Molecular profiling studies have identified several features associated with extramedullary growth. Integrating next-generation sequencing with single-cell analysis, Jelinek et al. found frequent co-occurrence of 1 q21 gain or amplification and MAPK-pathway mutations in EMD. CXCR4 expression was reduced, consistent with diminished dependence on CXCL12-mediated BM retention. Extramedullary PCs also showed lower expression of several established therapeutic antigens, including CD38, SLAMF7, GPRC5D, and FCRH5, which may contribute to reduced sensitivity to antibody-based and T-cell-redirecting therapies. In contrast, EZH2 and CD70 were upregulated and were proposed as potential alternative targets. CD8^+^ T cells and NK cells were the main effector populations detected within EMD tumors, indicating that immune cells remain present despite ineffective tumor control [47].

John et al. examined the organization of EMD lesions using RNA tomography, 10x Visium spatial transcriptomics, and scRNA-seq across 14 extramedullary biopsies. Individual lesions showed substantial regional heterogeneity with different areas characterized by distinct programs of proliferation, differentiation, immune reaction, and metabolism. Oxidative phosphorylation (OXPHOS) was prominent in several tumor regions suggesting metabolic adaptation of malignant PCs to growth outside of the BM. The immune compartment was characterized by T-cell exhaustion and impaired immune activation. Functionally competent T cells could still be detected but spatially confined to niches segregated from malignant PCs. In one patient responding to bispecific antibody treatment, changes in this organization were associated with treatment response. BCMA (TNFRSF17) and GPRC5D expression also varied across different areas of the same lesion, indicating that antigen-low populations may be missed when tissue is assessed only at bulk level [48].

Further comparison of EMD lesions with paired BM samples demonstrated clear differences between extramedullary and medullary immune environments [49]. EMD lesions were comparatively immune-poor and showed enrichment of CD16-negative NK cells with lower cytotoxic potential. Exhausted CD8^+^ T-cell populations were also increased, and cell–cell interaction analyses suggested that local interactions within the lesion contribute to the development of T-cell dysfunction [49].

These studies support the view that EMD differs from medullary MM at several biological levels. Changes in tumor cell signaling and antigen expression occur together with altered immune composition and reduced contact between effector cells and malignant PCs. These features may help explain the poor treatment responses observed in EMD and provide a rationale for investigating alternative therapeutic targets including CD70 and EZH2, as well as approaches aimed at restoring local immune activity.

## 6. Circulating Tumor Cells and Liquid Single Cell Profiling

### 6.1. The Evidence: Liquid Single Cell Profiling of Myeloma

MM has traditionally been studied using BM aspirates and biopsies, which remain central for diagnosis, risk stratification, and response assessment. Circulating tumor cells (CTCs) in myeloma offer a complementary approach. Obtained from peripheral blood, they provide a minimally invasive tool for assessing tumor burden, clonal composition, dissemination capacity, and disease evolution over time.

The clinical relevance of CTCs in MM has been recognized for decades. Early studies showed that both the presence and number of clonal CTCs are associated with advanced disease and shorter survival [50,51]. Studies using sensitive multiparameter and next-generation flow cytometry confirmed association between higher CTC levels, high-risk disease features and adverse outcome [52,53,54]. CTCs are not merely a measure of tumor burden but may also reflect biological properties related to marrow egress, dissemination, and aggressive behavior.

Single-cell analyses showed that CTCs recapitulate the genomic landscape found in paired BM MM cells. In some cases, mutations could be detected in blood with greater sensitivity, while single-CTC RNA sequencing enabled molecular classification and assessment of prognostic gene expression [55]. These findings were subsequently confirmed in independent analyses [56]. Later, Garcés et al. used next-generation flow cytometry to isolate matched CTCs and BM MM cells and found high concordance in chromosome arm-level copy-number alterations, including high-risk abnormalities. In patients with disease distributed across several anatomical sites, including extramedullary plasmacytomas, blood samples contained mutations derived from spatially separated clones [57]. CTCs may therefore provide information from more than one disease site. Transcriptional studies have also identified changes associated with PC egress from BM and circulation in peripheral blood [58].

Whole-genome approaches have extended these observations across different disease stages. The MinimuMM-seq workflow applied whole-genome sequencing to highly enriched CTCs across 261 individuals spanning MGUS, SMM, and MM spectrum. Tumor cells were recovered from approximately five of every six cases, allowing detection of translocations, copy-number changes, and changes in clonal composition. The circulating cells carried the same disease-defining lesions as the paired BM samples, and serial sampling captured clonal evolution over time [59].

SWIFT-seq further expanded the use of liquid single cell profiling by enabling detailed analysis of the small number of malignant PCs present in blood [60]. The study combined scRNA-seq together with B-cell-receptor sequencing in paired BM and CTC samples from 101 patients and healthy donors across the precursor and active disease stages. The approach enabled sequencing-based enumeration of CTCs, inference of cytogenetic abnormalities, estimation of tumor proliferative capacity, and longitudinal assessment of clonal dynamics. CTCs recapitulated the genomic complexity detected in the BM counterparts. Moreover, expression analysis identified a transcriptional signature of “circulatory capacity” that correlated with CTC burden, cytogenetic abnormalities, and shorter overall survival [60].

Fokkema et al. reported a different interpretation of the relationship between circulating and BM MM cells [61]. Using single-cell transcriptomics and whole-genome sequencing of matched BM and blood from newly diagnosed patients, they found that circulating and marrow PCs were transcriptionally similar, including at the level of individual clones. These data did not support the presence of a distinct transcriptional state that uniquely defines circulating PCs. Instead, high CTC counts were associated with tumor burden and proliferation, the MAF subgroup, and adverse secondary abnormalities such as 1 q gain or amplification, biallelic TP53 loss, and APOBEC mutational activity [61]. The clinical relevance of CTC burden was further demonstrated in the PERSEUS trial, where elevated CTC levels were associated with lower rates of measurable-residual-disease (MRD) negativity and shorter progression-free survival regardless of ISS, cytogenetic risk, LDH level, and assigned treatment [62].

CTC analysis may complement BM sampling in several clinical settings. CTC burden has prognostic value, while genomic and single-cell approaches can provide information on cytogenetic abnormalities, clonal evolution, antigen expression, and transcriptional states. The possibility of serial blood samplings is particularly attractive for monitoring disease during therapy, including proteasome inhibitors, anti-CD38 antibodies, CAR T-cell therapy, and bispecific antibodies. CTC profiling can also be combined with cell-free DNA analysis, where whole-exome sequencing of cell-free DNA and CTCs captures the tumor genome non-invasively, although at bulk rather than single-cell resolution [63].

However, several limitations remain. CTCs are scarce in many patients, particularly in precursor disease and during deep responses and their detection depends on assay sensitivity, PC markers, sample handling, and enrichment strategy. CTC abundance also varies with disease stage, treatment, circadian factors, and anatomical distribution. Finally, not all disease sites contribute equally to the circulating compartment, so resistant clones confined to protected BM regions or extramedullary lesions may therefore remain undetected in peripheral blood. CTC profiling should consequently be viewed as complementary to BM sampling and spatial tissue analysis.

### 6.2. Circulating Tumor Cells in MM: Dissemination Biology or Integrated Disease Biomarker?

It remains unclear whether CTCs represent a biologically distinct subpopulation with specific dissemination-associated properties, or mainly serve as a liquid biomarker of advanced, spatially complex, high-risk disease? Transcriptional programs associated with marrow egress to circulation suggest that some CTCs acquire features that facilitate dissemination [55,58], and SWIFT-seq identified a “circulatory-capacity” signature [60]. However, the close transcriptional similarity between CTCs and matched marrow myeloma cells suggests that their presence is an emergent feature of high-burden, biologically aggressive disease [61].

One possible explanation is that CTC number and CTC state reflect different aspects of disease biology. High CTC counts may largely reflect tumor burden, proliferation, vascular access, and high-risk genomic features. In contrast, transcriptional changes detected in circulating cells may be transient adaptations that support marrow egress and survival in the circulation, rather than stable properties of a distinct disseminating subclone.

From this perspective, CTCs are unlikely to represent a uniform population. Circulating cells may originate from heavily involved BM regions, focal or extramedullary lesions, or subclones with active dissemination programs. The relative contribution of these sources may differ across disease stages and treatment settings. This could explain why CTC burden has strong prognostic value even when circulating and BM PCs remain transcriptionally similar. A high CTC count may reflect aggressive disease without requiring the circulating cells to constitute a separate subtype. Whether a specific dissemination program exists independently of tumor burden and proliferation remains difficult to determine. Future studies should examine whether the circulatory-capacity signature remains significant after adjustment for these variables and whether they identify a distinct subclone or a reversible cellular state. Functional studies of intravasation, survival in circulation, and homing will also be required to determine the contribution of these transcriptional programs to dissemination.

Another important question concerns representativeness. Blood-based analysis may capture genetic alterations originating from anatomically separated BM and extramedullary clones and therefore provide broader molecular information than a single iliac crest aspirate [57]. However, this should be regarded as a biased multiregional readout rather than a comprehensive representation of the disease. Concordance between CTCs and BM is high for chromosome arm-level copy-number alterations, but lower for some translocations [57], and only clones that enter the circulation can be sampled. If CTC shedding is driven by proliferation, vascular proximity, and aggressive tumor behavior, then liquid profiling may preferentially detect the most active clones. In contrast, quiescent or drug-tolerant populations that persist through therapy within protective niches may be poorly represented. These could include OXPHOS-enriched PCs located near endosteal or vascular regions [64], locally restricted clones in focal or extramedullary lesions [48], and residual tumor cells supported by stromal niches after treatment [27]. CTC profiling may therefore reduce some of the sampling limitations of single site BM analysis, but it cannot eliminate spatial bias. Instead, it should be interpreted as a complementary liquid layer, combined with BM assessment, imaging of focal and extramedullary disease, and spatial tissue analysis.

The clinical meaning of elevated CTC levels remains to be clarified. High CTC burden is consistently associated with adverse outcome [62], yet it remains unclear whether CTCs actively contribute to progression or primarily reflect aggressive disease. If CTCs actively mediate dissemination, pathways involved in adhesion, trafficking, survival in the circulation, and homing may represent therapeutic targets. Conversely, if they primarily reflect tumor burden and genomic risk, their principal value may instead lie in risk stratification and longitudinal monitoring. These possibilities are not mutually exclusive and may vary between patients and disease stages.

This ambiguity is further underscored by the relationship between CTCs and cell-free DNA [63]. Cell-free DNA reflects total tumor-derived DNA, including DNA released by dying cells, whereas CTCs represent viable, intact tumor cells. Joint analysis could help distinguish viable circulating disease from broader tumor burden and treatment-induced tumor cell death.

From a clinical perspective, CTC analysis should not be used only as a numerical test. Enumeration by sensitive flow cytometry is clinically useful [54], but single-cell genomic and transcriptomic profiling, including whole-genome analysis of purified CTCs [59], can provide deeper insight on clonal structure, antigen expression, proliferative state, and treatment-resistant programs. This may be particularly important before and after T-cell-redirecting therapy, where CTCs could help monitor loss or downregulation of BCMA, GPRC5D, or other therapeutic targets, detect expansion of high-risk clones, and identify impending relapse before overt clinical progression.

## 7. Single-Cell Insights into Car T-Cell and T-Cell Engager Therapy: Response, Toxicity, Persistence and Resistance

T-cell-redirecting therapies have changed the treatment of relapsed and refractory MM (RRMM). BCMA-directed CAR T cells, including idecabtagene vicleucel and ciltacabtagene autoleucel, induce deep responses in heavily pretreated patients. Bispecific T-cell engagers (TCEs) targeting BCMA, such as teclistamab and elranatamab, GPRC5D with talquetamab, and FcRH5 with cevostamab have also shown substantial clinical activity [65,66,67,68,69]. Despite these advances, primary resistance and relapse remain common. Treatment outcome depends not only tumor antigen expression, but also on the functional state of the endogenous or engineered T cells, the surrounding BM microenvironment, and the ability of malignant PCs to adapt under immune pressure. Single-cell and multi-omic profiling have helped define these factors at cellular resolution.

In the CAR T-cell setting, single-cell profiling of blood and BM has shown that differences between responders and non-responders may already be present before infusion. Patients with early relapses or poor responses showed features of a more suppressive microenvironment, while inhibitory programs, including PD-1-associated dysfunction, were detected within the infused product [15]. A longitudinal single-cell atlas of 61 recipients of ide-cel or cilta-cel provided further evidence for differences in the responding T cell compartment. The higher complete response rate observed with cilta-cel, 78% versus 38% for ide-cel, was associated with the expansion of cytotoxic CD4^+^ T cells, whereas CD8^+^ T cells from non-responders showed weaker effector programs after infusion [70]. Thus, the differentiation and functional state of the CAR T cells appear to govern treatment efficacy.

The mechanism driving CAR T-cell dysfunction has been further defined in complementary single cell and functional analysis. In a cohort of 15 patients treated with four different CAR T-cell products, durable responders retained a higher proportion of persisting CD8^+^ effector-memory cells, whereas transient responders showed expansion of exhausted cytotoxic CD4^+^ CAR T cells. The BM microenvironment also contributed to this dysfunction. Nonclassical monocytes suppressed the killing capacity of CD4^+^ CAR T cells via TGF-β [71], showing that loss of CAR T-cell function can reflect both intrinsic T-cell changes and signals from surrounding myeloid cells.

Persistence is an additional factor of CAR-T functionality. Longitudinal single-cell multi-omic and gene-regulatory-network analysis of paired blood and BM samples showed that CAR T cells infiltrating the BM are more activated but also more exhausted than those in circulation. Poor persistence was associated with an altered transition from effector to memory states and accumulation of terminally differentiated cells, and a hyperexpanded IL10-expressing clone was identified in a patient with partial response [72].

Longitudinal profiling has also provided information on treatment-associated toxicity. In the same single-cel atlas, expansion of CD4^+^ cytotoxic T cells after cilta-cel was linked to both deeper response and immune-related toxicities [70]. Soluble BCMA levels decreased in parallel with CAR T-cell expansion and systemic inflammation, indicating that tumor debulking and the inflammatory toxicity of treatment arise from coordinated changes across CAR T cells, endogenous lymphocytes, and inflammatory myeloid compartments [70].

For bispecific engagers, which depend on endogenous T cells, the baseline state of the T cell compartment has particular relevance. scRNA-seq and T-cell-receptor sequencing of BM samples from patients treated with BCMA × CD3 engagers showed that treatment failure correlated with the accumulation of exhausted-like memory CD8^+^ T-cell clones expressing inhibitory receptors PD-1, TIGIT, and TIM-3 and showing reduced proliferative and cytokine responses. A low effector-to-tumor ratio and increased regulatory T cell abundance were also associated with poor response [73]. Similar findings were reported in the MajesTEC-1 study teclistamab, where response correlated with greater baseline T-cell fitness and a lower burden of exhausted and regulatory T cells [74].

Mechanistic and clinical observations suggest that deep response may rely on the expansion of selected cytotoxic T cell clones. Clonal CD8^+^ T-cell expansion accompanied a complete response to elranatamab [75], while repeated in vitro stimulation with a BCMA × CD3 engager resulted in robust expansion of only a subset of CD8^+^ T cells [76]. These findings support a role for both functional capacity and clonal composition of the CD8^+^ T-cell compartment in determining response to bispecific engagers.

Loss or alteration of the target antigen is an important mechanism of acquired resistance to both CAR-T cell and bispecific therapies. One of the earliest examples was identified by longitudinal single-cell genomic analysis of a patient who relapsed after BCMA CAR T-cell therapy with biallelic loss of TNFRSF17, the gene encoding BCMA [77]. Larger genomic studies subsequently showed that relapse after BCMA- or GPRC5D-directed therapy may result from focal biallelic deletion, mutation, or selective expansion of pre-existing antigen-deficient subclones [78]. Resistance to GPRC5D-directed engagers can arise through several mechanisms, including biallelic deletion, monoallelic loss combined with GPRC5D mutations or indels, and epigenetic silencing of regulatory regions [79]. Single-nucleus multi-omic analysis independently confirmed genetic and epigenetic inactivation of GPRC5D as mechanisms of acquired resistance [80].

Taken together, these studies identify a set of shared and interacting determinants. Future strategies will need to address both the malignant PC and the effector compartment, through dual or sequential antigen targeting, checkpoint modulation, myeloid-directed intervention, metabolic or cytokine support of T-cell function, and combinations with immunomodulatory drugs or CELMoDs.

## 8. Metabolic Reprogramming Across the Tumor, Immune, and Stromal Compartments

Metabolic reprogramming is now recognized as an important feature of MM BM, and single-cell and multi-omic technologies have reframed it from a cell-intrinsic property of the tumor into an organizing principle of the entire niche. MM cells reside in a spatially heterogeneous tissue in which oxygen tension, nutrient availability, and bone-remodeling signals differ across various regions. These local conditions can influence PC survival, immune escape, bone destruction, and therapy resistance.

The metabolic demands of malignant PCs increase with disease progression. scRNA-seq across MGUS, SMM, and active MM has shown progressive enrichment of OXPHOS and the anabolic MYC-target and mTORC1 programs [7]. Single-cell transcriptomic profiling has further identified MM cell subpopulations with increased proliferative capacity, high-copy number alteration scores, and enhanced glucose metabolic activity, linking glycolytic fitness of malignant PCs to aggressive disease states [81]. Integrated single-cell, bulk, and single-cell metabolic profiling similarly showed increased glycolysis, OXPHOS, and glutamine metabolism in malignant PCs, together with induction of effectors such as LDHA and GLS1 [82].

Hypoxia and nutrient deprivation impose additional pressures on MM cells and can alter their interaction with surrounding stromal cells. Under hypoxic and glucose-limited conditions, MM cells increase glycolytic activity and lactate production, resulting in acidification of the local milieu. At the same time, they rely on adaptive pathways that maintain ATP production, redox balance, and proteostasis [83,84]. Stromal cells can contribute to this adaptation. Solimando et al. described a form of transmitophagy using a co-culture system mimicking the hypoxic, nutrient-starved niche, in which dysfunctional mitochondria are transferred from MM cells to stromal cells for degradation. Mitochondria were also exchanged between stromal cells through tunneling nanotubes, and this process was associated with resistance to apoptosis [85].

Proteogenomic profiling further underscores the therapeutic importance of metabolism at the protein level. Malignant PCs display dysregulated protein and phosphoprotein networks that are only partially predicted by transcriptomic or genomic alterations. Mitochondrial, proteostasis, and stress response pathways appear to be frequently affected by post-transcriptional regulations, indicating that protein-level analysis may reveal vulnerabilities that are missed by transcriptomic profiling approaches [86].

Spatial studies suggest that tumor cell metabolism varies across BM. MM cells located near supportive stromal or immune niches may differ metabolically from those exposed to hypoxia, nutrient limitations, or high tumor density [45]. Imaging mass cytometry provided direct evidence for this organization, identifying PC neighborhoods with different metabolic features. One was enriched for OXPHOS and associated with focal growth and proximity to endothelial cells, whereas another showed a more glycolytic profile and greater interspersion of PCs with immune cells. Importantly, the extent of these spatial interactions correlated with shorter progression-free survival, emphasizing metabolic state as a spatially patterned and prognostically meaningful property of the niche [64].

BM adipocytes may also provide metabolic support to MM cells. Their abundance increases with age and may supply fatty acids (FA) and adipokine signals that favor malignant growth [87]. Recent work showed that MM cells promote lipolysis in BM adipocytes and take up the released FA, fueling energy production, membrane biosynthesis, and stress adaptation [88]. Lipid availability may also affect antitumor immunity, as long-chain FA in BM were shown to impair CD8^+^ T-cell metabolism and reduce mitochondrial mass and function. These changes were reversible in treatment-responsive patients but persisted in relapsed disease [89].

Glutamine metabolism provides another link between malignant PCs, immune cells and the BM compartment. MM cells consume large amounts of glutamine, potentially reducing its availability to neighboring lymphocytes. Single-cell profiling of BM and peripheral-blood T cells identified one mechanism of this restriction. Activation of the XBP1-dependent unfolded-protein-response (UPR) program reduced expression of the glutamine transporter SLC38A2 in cytotoxic CD8^+^ T cells, limiting glutamine uptake and promoting senescence, exhaustion, and impaired function [90].

Changes in amino acid availability may shape bone remodeling. High glutamine consumption by MM is accompanied by increased glutamate release, which promotes osteoclast differentiation via NF-κB–NFATc1 signaling, with EAAT1 serving as the glutamate transporter [91]. Consistent with the clinical relevance of this axis, integrative single-cell and bulk analyses of glutamine-metabolism-related genes have generated prognostic signatures that stratify survival and predict immune dynamics and drug sensitivity [92].

Finally, the metabolic state of the niche directly affects the response to T-cell-redirecting immunotherapy. Hypoxia and the MM microenvironment suppress mTOR activity and metabolic reprogramming in CD8^+^ T cells, reducing their response to BCMA × CD3 bispecific stimulation [93]. Glutamine availability is also important for CAR T-cell function. Navarro et al. showed that glutamine consumption by MM cells deprives BCMA-targeting CAR T cells of a nutrient and impairs their activity. Forced expression of the glutamine transporter ASCT2 in the CAR T cells restored mTORC1 signaling, oxidative and glycolytic output, and CAR T persistence, resulting in improved tumor control and longer survival in mouse models. In patient samples, higher ASCT2 expression by tumor cells was associated with poorer responses to combined immunotherapy and BCMA-directed CAR T-cell treatment [94].

## 9. Critical Evaluation of Spatial Platforms in MM

Spatial technologies are not interchangeable. They differ in molecular breadth, spatial resolution, tissue compatibility, segmentation strategy, and analytical output. These differences directly affect how the MM niche is interpreted. This is particularly important in MM because biologically relevant interactions occur across several spatial scales: whole marrow regions, focal lesions, stromal and vascular neighborhoods, immune-excluded plasma-cell clusters, and direct cell–cell contacts. No single platform captures all these layers equally well. Therefore, spatial findings in MM should be interpreted in relation to the strengths and limitations of the platform used.

Whole-transcriptome spatial profiling is represented by the original spatial transcriptomics approach and by Visium-based platforms, in which polyadenylated or probe-captured transcripts are barcoded on a spatially indexed array, yielding an unbiased, near-genome-wide expression readout across formalin-fixed, paraffin-embedded (FFPE) tissue sections [95,96]. It is especially valuable for discovery because it enables transcriptome-wide gene-expression analysis while preserving tissue architecture [37]. The strengths of Visium for MM are therefore hypothesis generation, cross-compartment discovery, and identification of previously uncharacterized immune, inflammatory, and metabolic programs in intact bone marrow sections. Its major limitations are the sub-cellular ambiguity of the 55 μm spot, which mixes plasma cells with adjacent T cells, macrophages and mesenchymal stromal cells. Sensitivity per cell is also lower than that of imaging-based platforms, and mineralized BM trephines demand careful decalcification protocols to preserve RNA integrity [97]. Muiños-Lopez et al. specifically noted several challenges relevant to BM spatial transcriptomics, including decalcification-related RNA damage, low cellularity of non-hematopoietic compartments, lack of single-cell resolution in current Visium-based approaches, sectioning artifacts, and dependence on matched single-cell references for annotation [45].

Single-cell and subcellular in situ transcriptomic platforms, such as Xenium and CosMx Spatial Molecular Imager (SMI), address several of these limitations. The CosMx SMI platform belongs to the family of imaging-based, in situ hybridization technologies that resolve individual transcripts and assign them to individual cells through membrane- and nucleus-based segmentation [40]. The original 980-plex assay has been extended to 6000-target and human whole-transcriptome panels, retaining sensitivity of one to two copies per cell and false-call rates below 0.01 counts per cell [40,96]. Comparable imaging platforms, 10x Genomics Xenium and Vizgen MERSCOPE, share the single-cell and even subcellular paradigm and are frequently benchmarked alongside CosMx [95,96]. CosMx and MERSCOPE are more effective in detecting low-level mRNA compared to Xenium, which can examine larger tissue areas and directly obtain precise spatial information. In MM, this level of resolution is essential for determining intra-tumoral antigen heterogeneity, at the level of individual malignant PCs, an increasingly urgent question in an era of BCMA- and GPRC5D-directed bispecific and CAR-T therapies, where cell-level rather than region-level antigen loss drives relapse. These imaging platforms also provide direct visualization of T-cell exclusion versus infiltration, and, in the mineralized BM context, allow endosteal, perivascular and central marrow niches to be distinguished at cellular resolution [40,97]. Recent MM work illustrates this advantage. Yip et al. used Xenium-based spatial transcriptomics in human BM trephine biopsies and showed that optimized tissue processing allowed resolution of major hematopoietic, stromal, vascular, and PC populations in preserved BM architecture [13]. The trade-off is that these high-resolution in situ platforms are panel-based. Discovery of novel spatially variable programs is constrained to the probes included, and cell segmentation in the highly cellular, morphologically heterogeneous BM remains a recognized technical challenge that can propagate into cell-type miscalling [96,97]. This is a critical issue in MM because important biology may involve unexpected metabolic, stromal, inflammatory, or resistance-associated programs. Thus, single-cell and subcellular spatial platforms are strongest when used to validate and refine hypotheses generated by whole-transcriptome spatial profiling or scRNA-seq.

The GeoMx DSP platform occupies an intermediate position. Rather than tiling the entire tissue section, it uses fluorescent morphology markers to define user-selected areas of illumination (AOIs), from which UV-cleavable oligonucleotide tags conjugated to antibody or in situ hybridization probes are released and quantified by nCounter or next-generation sequencing [38]. Both a Whole Transcriptome Atlas (~18,000 targets) and targeted protein panels can be interrogated on the same FFPE slide, allowing paired transcriptomic and proteomic characterization of morphologically defined MM compartments [38,95]. In MM, GeoMx DSP has been applied to formalin-fixed BM biopsies from patients with MGUS, SMM, and active MM to characterize T-cell excluded regions and to identify CLEC9A+ dendritic-cell portals of antigen-specific T-cell entry, a spatial architecture with direct implications for CAR T-cell and bispecific antibody response [98]. Compartment-resolved GeoMx DSP profiling has also been used in paired pre- and post-daratumumab BM biopsies to demonstrate that acquired resistance is associated with a spatially restricted reduction in immune cell numbers within tumor centers, in parallel with an enrichment of CD8^+^ T-cell exhaustion and NK dysfunction signatures [99]. The main limitation of GeoMx DSP is that its standard output represents an average signal from selected regions rather than individual cells [38]. Therefore, it is powerful for comparing spatial compartments, but less suited for providing cellular-resolution interpretations such as direct cell–cell contacts or intralesional antigen heterogeneity. In MM, GeoMx can define regional transcriptional or proteomic states, but it should ideally be paired with single-cell spatial imaging or multiplexed protein imaging to assign those states to specific cellular populations.

Multiplexed protein imaging platforms, including CODEX and imaging mass cytometry, provide a complementary layer. Imaging mass cytometry combines antibody staining with mass cytometry-based detection to measure multiple proteins in tissue sections while preserving spatial organization [100]. CODEX uses oligonucleotide-conjugated antibodies and cyclic imaging to generate highly multiplexed protein maps of intact tissues [101]. This is particularly important in MM because many clinically relevant states are defined at the protein level, including therapeutic antigen expression, immune checkpoint expression, cytotoxic effector phenotype, macrophage polarization, NK cell phenotype, proliferation, and phospho-signaling. In normal human bone marrow, CODEX combined with single-cell transcriptomics has been used to map BM spatial architecture and define organized marrow niches [42]. In MM, imaging mass cytometry has revealed immune-metabolic PC neighborhoods linked to disease progression, bone disease, and progression-free survival [64].

Together, these platforms answer different but complementary questions. In MM, the strongest biological interpretation comes from integrating these layers rather than relying on a single platform (Table 1).

## 10. Hematopoietic Stem Cell Transplantation as a Modulator of the MM Immune Microenvironment: Opportunities for Precision Immunotherapy

MM progression reflects both tumor-intrinsic changes and alterations in the BM microenvironment, including immune dysfunction, stromal support, spatial heterogeneity, metabolic adaptation, and therapy-driven selection. HSCT is therefore relevant not only for its cytoreductive effect, but also because it may alter the cellular context in which residual disease persists.

Autologous HSCT remains a standard component of treatment for eligible patients with newly diagnosed MM. High-dose melphalan reduces tumor burden and produces profound lymphodepletion, followed by hematopoietic and immune reconstitution. This sequence creates a period in which residual malignant PCs are reduced, and immune populations begin to recover [102]. The post-transplant state may therefore differ substantially from the pre-treatment BM environment, although recovery is not necessarily complete.

Clinical studies support this view. Chen et al. reported improved response depth after autologous transplantation alongside recovery of B- and T-cell populations, while the CD4/CD8 ratio remained altered and NK-cell numbers were reduced early after transplant [103]. Parmar et al. showed that patterns of immune reconstitution were associated with clinical outcome, with persistent T-cell dysfunction and exhaustion-like features linked to poorer prognosis [104]. The maturation state of recovering NK cells has also been associated with outcome [19]. These findings indicate that post-HSCT immune recovery is heterogeneous and clinically meaningful, but it does not necessarily restore a fully normal immune state.

Single-cell approaches have provided a more detailed view of this process. Li et al. used BM scRNA-seq in patients treated with high-dose melphalan followed by HSCT and observed a marked reduction in malignant PCs together with hematopoietic and immune recovery, yet small residual malignant populations remained detectable [105]. In paired pre- and post-transplant BM samples, scRNA-seq and T-cell receptor sequencing showed that disease progression was associated with enrichment of exhausted CD8^+^ T cells, increased stromal interactions, reduced T-cell receptor diversity, and loss of CD56^bright^ NK cells and plasmacytoid DCs [106].

Long-term studies suggest that the consequences of MM can persist for years. Lutz et al. characterized BM from long-term survivors up to 17 years after first-line therapy and found that even patients without detectable residual disease retained sustained immune alterations, a state described as “immunological scarring” [107]. Similar persistence has been reported in the stromal compartment. De Jong et al. identified a myeloma-associated inflammatory mesenchymal stromal cell state that remained detectable after induction therapy despite reduction in tumor burden. These observations suggest that treatment can effectively deplete malignant PCs without fully restoring the non-malignant BM niche to its normal state [27]. The goal, therefore, should not be to assume complete immune normalization after transplantation, but to define which immune compartments recover, which remain dysfunctional, and how these patterns influence relapse risk.

This has direct implications for current immunotherapeutic strategies. The post-HSCT period may provide an important therapeutic window: reduced tumor burden, lymphodepletion, and immune rebuilding may improve the conditions for immune-based consolidation. At the same time, persistent T-cell exhaustion, myeloid suppression, delayed CD4^+^ recovery, impaired NK-cell activity, or residual spatially protected tumor clones may reduce the effectiveness of post-transplant immunotherapy. Single-cell and spatial profiling could help identify patients with favorable immune reconstitution and those who require additional immune modulation before or alongside immunotherapy.

Longitudinal studies will be important for defining these differences. Sampling before conditioning, immediately before graft infusion, after engraftment, during maintenance therapy, and at relapse, could be used to follow changes in malignant PCs, T cell states, NK cell function, myeloid populations and stromal compartment over time. Spatial studies could then determine whether residual malignant PCs persist in protected stromal, vascular, or immune-excluded marrow regions after transplantation. Longitudinal profiling may clarify whether immune normalization after HSCT enhances the efficacy of CAR T cells, T-cell engagers, CELMoDs, or checkpoint-modulating strategies. Finally, liquid single-cell profiling of CTCs may help detect residual high-risk clones not captured by a single BM biopsy. In conclusion, HSCT may contribute to partial remodeling of the MM BM immune microenvironment by reducing tumor burden and enabling hematopoietic and immune reconstitution, but the available data indicate that this process is incomplete and heterogeneous.

## 11. Rethinking Immune Remodeling and Therapeutic Readiness in MM

Immune dysfunction is generally thought to increase as MM progresses from MGUS to symptomatic disease, and several studies have linked the degree of immune disturbance with worse outcome [9,10,14]. This is consistent with the impaired cytotoxic activity, increased susceptibility to infections, and diminished response to immune-based therapy seen in advanced MM. However, several observations challenge this linear interpretation. In high-risk SMM, patients whose immune profiles most closely resembled those of healthy donors fared worse, whereas some degree of immune remodeling was associated with better responses to immunotherapy [108]. These findings suggest that deviation from a healthy immune profile does not necessarily indicate ineffective antitumor immunity.

**Figure 2:**
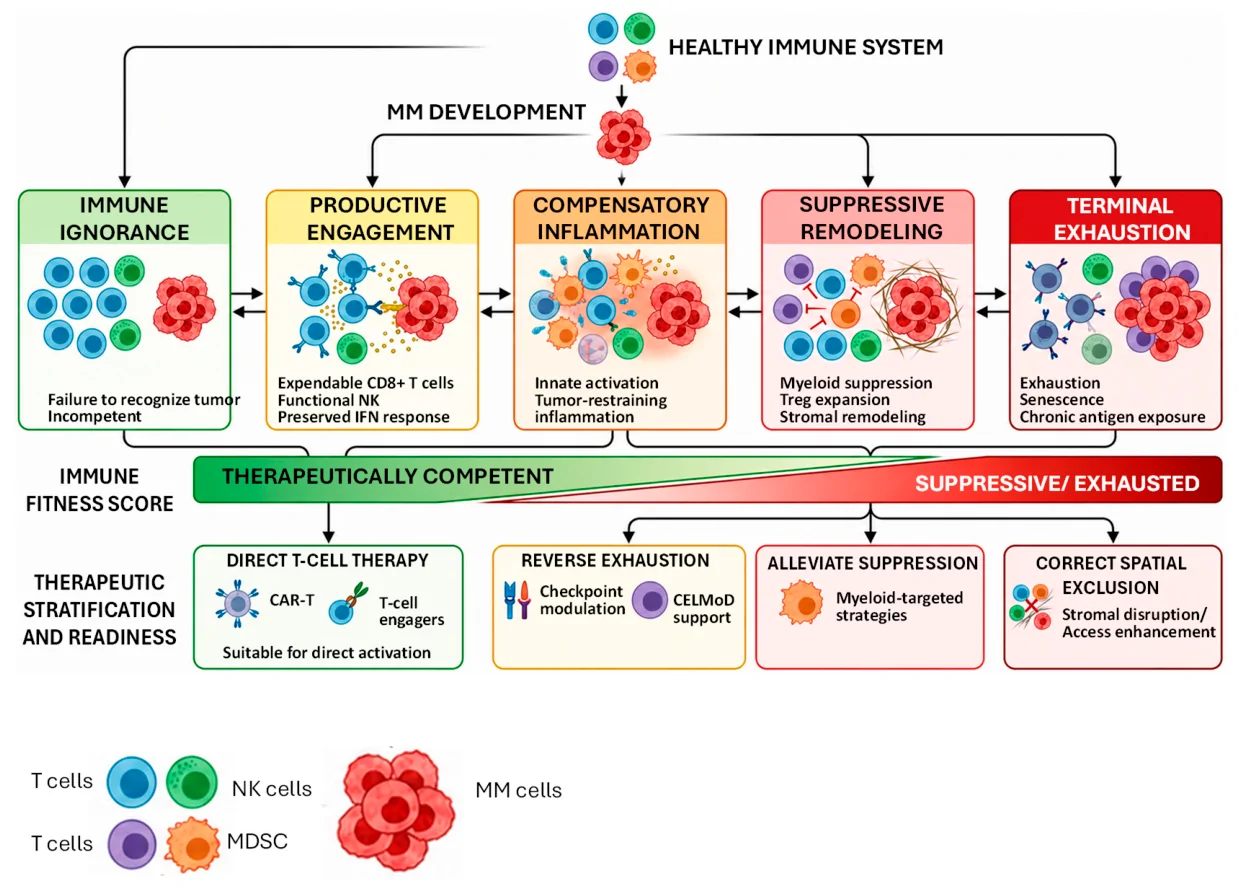
Conceptual framework of immune remodeling, immune fitness, and therapeutic readiness during multiple myeloma development. Schematic representation of the dynamic immune states that may emerge during multiple myeloma (MM) development and progression, and their potential implications for immune-directed therapy. The model depicts a continuum of partially overlapping and potentially reversible immune states shaped by interactions between malignant plasma cells and the bone marrow microenvironment. A healthy immune compartment can evolve toward distinct patterns of tumor recognition, inflammatory activation, immune suppression, and dysfunction as MM develops. In immune ignorance, immune populations may remain numerically present and appear relatively preserved but fail to effectively recognize or respond to malignant plasma cells, resulting in limited antitumor activity. Productive engagement represents a therapeutically favorable state characterized by effective tumor recognition, expandable cytotoxic CD8^+^ T-cell populations, preserved natural killer (NK)-cell function, and an active interferon-associated response capable of supporting antitumor immunity. With persistent tumor exposure, compensatory inflammation may emerge, characterized by activation of innate immune populations and inflammatory programs that can initially contribute to tumor restraint but also reflect sustained antigenic stimulation. Further disease-associated remodeling may lead to a suppressive state, in which myeloid-mediated suppression, expansion of regulatory T cells (Tregs), and remodeling of the stromal and extracellular-matrix compartment progressively restrict effective immune-cell function and tumor accessibility. At the dysfunctional end of the continuum, terminal exhaustion is characterized by profound loss of immune effector capacity associated with chronic antigen exposure, T-cell exhaustion, and senescence-like cellular states. Different immune states may require distinct therapeutic strategies. Patients with relatively preserved immune fitness may be suitable for direct T-cell–redirecting approaches, including chimeric antigen receptor (CAR)-T-cell therapy and T-cell engagers. Where immune dysfunction is dominated by exhaustion, therapeutic approaches aimed at restoring effector-cell fitness, including checkpoint-pathway modulation or cereblon E3 ligase modulator (CELMoD)-mediated immune support, may be required. Microenvironments dominated by suppressive myeloid populations may benefit from myeloid-targeted strategies, whereas tumors characterized by stromal or extracellular-matrix-mediated immune exclusion may require disruption of spatial barriers or enhancement of immune-cell access. Collectively, the model proposes that assessment of functional immune fitness and spatial microenvironmental organization may complement conventional measures of tumor burden and immune composition when stratifying patients for immune-directed treatment. Abbreviations: MM, multiple myeloma; CD8^+^ T cell, cytotoxic T lymphocyte; NK, natural killer; IFN, interferon; Treg, regulatory T cell; CAR-T, chimeric antigen receptor T-cell therapy; CELMoD, cereblon E3 ligase modulator.

A relatively preserved immune composition in high-risk precursor disease may, in some cases, reflect limited recognition of the malignant clone rather than effective immune control. By contrast, changes in immune cell compartment or activation may indicate that an antitumor response has been already initiated. The biological consequences of this response are likely to depend on its duration and functional quality. Early recognition may promote expansion of cytotoxic or clonally reactive T cells, activation of innate immune populations, and inflammatory responses capable of restricting tumor growth. With increasing tumor burden and prolonged antigen exposure, the same response may become progressively impaired through T cell exhaustion, senescence, suppressive myeloid signaling, stromal changes and other mechanisms that limit immune function. If tumor control ultimately fails, sustained antigen exposure, rising tumor burden, stromal remodeling, and suppressive myeloid signaling may gradually shift this response toward exhaustion, senescence, and functional paralysis. Immune remodeling is therefore better viewed as a dynamic continuum from recognition through activation toward terminal dysfunction than as a monotonic decline from health (Figure 2).

For this reason, the functional state of the immune compartment may be more informative than its similarity to healthy marrow. Features such as expandable cytotoxic T-cell clones, a preserved interferon response, functional NK cell activity, and effective antigen presentation may indicate retained antitumor capacity. In contrast, accumulation of exhausted CD8^+^ T cells, regulatory T cells, suppressive myeloid populations, or dysfunctional DCs is more likely to reflect impaired immune control. This distinction is decisive in the era of CAR T cells and T-cell engagers, whose efficacy depends not on tumor antigen expression alone but on the presence of effector cells capable of activation, expansion, and sustained cytotoxicity [73,74]. Furthermore, immune state must be interpreted together with tissue localization. The presence of cytotoxic lymphocytes in a BM sample does not necessarily imply effective tumor control if these cells are excluded from malignant PC-rich regions or concentrated in areas dominated by suppressive stromal or myeloid signals.

Comparison with healthy BM remains useful, but it may be insufficient for predicting disease course or response to immunotherapy. A more informative approach might be instead to characterize functional states: immune ignorance, productive engagement, compensatory inflammation, suppressive remodeling, and terminal exhaustion. Such a framework could help explain why some patients with precursor disease remain stable whereas others progress despite an apparently preserved immune composition, and why some patients respond to immunotherapy despite evidence of immune activation. It would also distinguish patients who require immune stimulation from those who would benefit instead from reversal of exhaustion, alleviation of suppression, or correction of spatial immune exclusion. Therefore, we suggest replacing “immune abnormality” scores, which primarily measure deviation from healthy marrow, with “immune-fitness” scores. Importantly, these features should be interpreted in relation to their spatial position, not only their abundance (Figure 2).

This functional stratification could guide the selection and optimization of T-cell-redirecting therapy. Patients with preserved immune fitness may be suitable for direct treatment with CAR T cells or T-cell engagers. In contrast, patients with dominant T-cell exhaustion may require checkpoint modulation or CELMoD support. Those with myeloid suppression may benefit from myeloid-targeting strategies, and those with spatial immune exclusion may require approaches that disrupt stromal barriers or improve effector-cell access to tumor-rich regions. This is consistent with evidence that baseline T-cell fitness is associated with response to T-cell engagers [73,74].

It would also reframe therapy timing as an immunological decision. Immunotherapy may be most effective during a window of active immune engagement. This may occur in high-risk smoldering disease, early relapse, post-transplant immune reconstitution, or low-burden MRD, before chronic antigen exposure drives terminal exhaustion.

## 12. Conclusions

Single-cell, spatial, and multi-omic studies have expanded the view of MM beyond the malignant PC itself. Disease progression, therapeutic response, and relapse are not determined by malignant PC genetics alone. Rather, they are shaped by where tumor cells reside, which stromal and immune cells surround them, which metabolic resources are available, and how these interactions evolve under treatment pressure.

Focal lesions, extramedullary sites, and protected residual niches may harbor tumor cell populations with distinct transcriptional states and different interactions with surrounding cells. Integrating tissue-based profiling with imaging and blood-based approaches may therefore provide a more complete assessment than any single method alone.

The immune compartment also requires functional rather than purely descriptive interpretation. A healthy-like immune profile may reflect immune ignorance, whereas a remodeled immune compartment may indicate tumor recognition and partial antitumor engagement. Future biomarkers should move beyond descriptive immune abnormality scores toward functional immune fitness scores that integrate cytotoxic T cell reserve, NK cell function, antigen presentation, myeloid suppression, exhaustion, and spatial access to tumor cells.

CTCs add a complementary source of information on tumor burden, clonal evolution, and dissemination. However, blood-based profiling cannot capture populations that remain confined to protected BM or extramedullary sites. CTC analysis is therefore most useful when interpreted together with BM assessment and imaging, particularly in patients with spatially heterogeneous disease.

Autologous HSCT further illustrates the importance of considering the non-malignant BM compartment. HSCT reduces tumor burden and initiates immune reconstitution that may influence the fate of residual disease. However, current evidence indicates that immune and stromal normalization after transplantation is incomplete and heterogeneous. Consequently, the post-transplant period may represent an important therapeutic window, but only if immune recovery is functionally competent and residual disease is not protected within resistant spatial niches.

The next challenge is to translate these insights into clinically useful tools. Future studies should be longitudinal, spatially resolved, and clinically annotated. They should profile patients before treatment, during response, at minimal residual disease, after transplantation, and at relapse. Such studies should integrate malignant PC states with immune competence, stromal persistence, metabolic adaptation, antigen expression, CTC dynamics, and spatial localization of residual disease.

## Figures and Tables

**Table 1:** Critical comparison of the principal spatial platforms used to study the MM niche.

Platform	Spatial Unit	Molecular Breadth	Sensitivity	Tissue Compatibility	Key Value in MM	Main Limitation	Representative References
Visium/Visium HD (10x Genomics)	55 μm spot (Visium); 2 μm capture grid, effective analysis at 8–16 μm bins (Visium HD)	Whole transcriptome (unbiased); ~18,000 protein-coding genes captured per spot	Moderate; spot-averaged (~500–2000 UMIs per Visium spot)	FFPE and fresh-frozen; requires optimized decalcification and preserved RNA integrity (DV200) for BM trephines	Unbiased discovery of spatially variable programs in intact BM and extramedullary lesions; maps PC-rich regions, stromal, inflammatory, and metabolic programs	Sub-cellular ambiguity of the 55 μm spot (multiple cells per spot, particularly in densely packed BM); cell-type deconvolution requires a paired scRNA-seq reference	[37,45,48]
Xenium In Situ (10x Genomics)	Individual cell and subcellular (~200 nm transcript localization); padlock probes + rolling-circle amplification	Targeted panels of ~300–1000 genes (standard); Xenium Prime panel (5001 genes)	High; false-call rate < 0.1%; sensitivity varies by gene and panel design	FFPE and fresh-frozen; validated on BM trephines with optimized EDTA decalcification	Cellular-resolution mapping of malignant and normal PCs, stromal, vascular, and immune populations in preserved BM architecture; visualization of T-cell exclusion and cellular-level antigen heterogeneity	Panel-based; biological interpretation constrained to selected probes; cell segmentation remains challenging in densely packed BM	[13,39]
CosMx SMI (NanoString/Bruker)	Individual cell and subcellular (~50 nm transcript localization); cyclic in situ hybridization with fluorescent barcodes	Targeted panels of ~1000 (standard) to ~6000 targets; whole-transcriptome panel emerging	High; single-transcript detection at 1–2 copies per cell; false-call rate ~0.01 counts per cell	FFPE and fresh-frozen	Direct cellular-level testing of intra-tumoral antigen heterogeneity and T-cell/plasma-cell proximity, directly relevant to CAR-T and bispecific-antibody biology	Panel-based; cell segmentation in densely packed BM propagates into cell-type miscalling; higher instrument and reagent cost	[40]
ROI-based GeoMx DSP (NanoString/Bruker)	User-selected area of illumination (AOI); typically multicellular (~50–500 μm); can approach single-cell dimensions with reduced throughput	Whole Transcriptome Atlas (~18,000 targets) or Cancer Transcriptome Atlas (~1800 targets); concurrent protein panels (up to ~100 markers across combined modules); RNA and protein readouts on the same slide	High within selected AOIs; nCounter or NGS readout	FFPE (well-suited to archival BM trephines) and fresh-frozen	Compartment-resolved comparison of CD138+ tumor centers and CD3 + /CD45+ immune margins; dissects T-cell exclusion, antigen-specific T-cell entry, and monoclonal-antibody (daratumumab) resistance mechanisms	ROI selection is investigator-driven and hypothesis-dependent; regions outside AOIs are not sampled; not truly single-cell resolution by default	[38,98,99]
Multiplexed protein imaging: CODEX	Individual cell (~250 nm optical resolution); DNA-barcoded antibodies + cyclic fluorescence imaging	Targeted protein panel of 40–60 markers per section	High per marker; iterative signal cycling	FFPE and fresh-frozen	Maps organized BM biogeography; validates immune-cell architecture, therapeutic-antigen expression, and macrophage/stromal neighborhoods in the MM niche	Panel-based; long acquisition times due to cyclic imaging; lower molecular breadth than transcriptome-wide platforms	[42,101]
Imaging Mass Cytometry (IMC) (Hyperion)	Subcellular (~1 μm laser-ablation spot); metal-tagged antibodies + CyTOF detection	Targeted protein panel of up to ~45 markers per section	High; no spectral overlap between metal isotopes	FFPE and fresh-frozen	Reveals immune-metabolic PC neighborhoods, distance-to-bone gradients, and cellular states linked to disease progression, bone disease, and progression-free survival	Panel-based; specialized instrumentation; slower acquisition than fluorescence-based multiplexed imaging	[64,100]

Abbreviations: AOI, area of illumination; BCMA, B-cell maturation antigen; BM, bone marrow; CAR-T, chimeric antigen receptor T cell; CD, cluster of differentiation; CODEX, co-detection by indexing; CosMx SMI, CosMx Spatial Molecular Imager; DSP, Digital Spatial Profiler; EDTA, ethylenediaminetetraacetic acid; FFPE, formalin-fixed paraffin-embedded; GPRC5D, G-protein–coupled receptor family C group 5 member D; IMC, imaging mass cytometry; MM, multiple myeloma; NGS, next-generation sequencing; PC, plasma cell; ROI, region of interest; UMI, unique molecular identifier.

## Data Availability

No new data were created or analyzed in this study. Data sharing is not applicable to this article.
